# *egr3* is a mechanosensitive transcription factor gene required for cardiac valve morphogenesis

**DOI:** 10.1126/sciadv.adl0633

**Published:** 2024-05-15

**Authors:** Agatha Ribeiro da Silva, Felix Gunawan, Giulia L. M. Boezio, Emilie Faure, Alexis Théron, Jean-François Avierinos, SoEun Lim, Shivam Govind Jha, Radhan Ramadass, Stefan Guenther, Mario Looso, Stéphane Zaffran, Thomas Juan, Didier Y. R. Stainier

**Affiliations:** ^1^ Max Planck Institute for Heart and Lung Research, Department of Developmental Genetics, Bad Nauheim, Germany.; ^2^ German Centre for Cardiovascular Research (DZHK), Partner Site Rhine-Main, Bad Nauheim, Germany.; ^3^ Cardio-Pulmonary Institute (CPI), Bad Nauheim, Germany.; ^4^ Aix Marseille Université, INSERM, MMG, U1251, 13005 Marseille, France.; ^5^ Service de Chirurgie Cardiaque, AP-HM, Hôpital de la Timone, 13005 Marseille, France.; ^6^ Service de Cardiologie, AP-HM, Hôpital de la Timone, 13005 Marseille, France.; ^7^ Bioinformatics and Deep Sequencing Platform, Max Planck Institute for Heart and Lung Research, Bad Nauheim, Germany.; ^8^ Bioinformatics Core Unit (BCU), Max Planck Institute for Heart and Lung Research, Bad Nauheim, Germany.

## Abstract

Biomechanical forces, and their molecular transducers, including key mechanosensitive transcription factor genes, such as *KLF2*, are required for cardiac valve morphogenesis. However, *klf2* mutants fail to completely recapitulate the valveless phenotype observed under no-flow conditions. Here, we identify the transcription factor EGR3 as a conserved biomechanical force transducer critical for cardiac valve formation. We first show that *egr3* null zebrafish display a complete and highly penetrant loss of valve leaflets, leading to severe blood regurgitation. Using tissue-specific loss- and gain-of-function tools, we find that during cardiac valve formation, Egr3 functions cell-autonomously in endothelial cells, and identify one of its effectors, the nuclear receptor Nr4a2b. We further find that mechanical forces up-regulate *egr3*/*EGR3* expression in the developing zebrafish heart and in porcine valvular endothelial cells, as well as during human aortic valve remodeling. Altogether, these findings reveal that EGR3 is necessary to transduce the biomechanical cues required for zebrafish cardiac valve morphogenesis, and potentially for pathological aortic valve remodeling in humans.

## Teaser

*EGR3*, a mechanosensitive transcription factor gene in zebrafish and mammals, is necessary for cardiac valve morphogenesis.

## INTRODUCTION

Tissue morphogenesis is intrinsically linked to the mechanical forces that are required for the cellular and molecular processes defining cell fate (*1*–*4*). In this context, endothelial cells, which line the blood vessels throughout the body, are specialized according to the function and mechanical properties of the tissues they reside in as well as blood flow patterns (*5*–*8*). During heart development, the specialized endothelial cells lining the cardiac wall, known as endocardial cells (EdCs), invade the adjacent extracellular matrix (ECM) in the atrioventricular (AV) canal and outflow tract (OFT) in response to oscillatory shear stress (*9*–*11*). These migrating EdCs acquire mesenchymal characteristics, differentiate into valve interstitial cells (VICs), and proliferate to give rise to the valve leaflets (*11*–*21*), which are essential for cardiac function as they guarantee unidirectional blood flow.

Extensive work in vertebrates has shown that perturbation of mechanical forces in the heart severely affects valve formation (*10*, *22*–*25*). In particular, the zebrafish model allows for the observation and manipulation of intracardiac mechanical forces while imaging valve morphogenesis at single-cell resolution. For example, stopping cardiac contraction, and consequently blood flow, results in a valveless phenotype (*26*). Molecularly, the expression of several genes has been shown to be flow sensitive (*11*, *27*), and the classical endothelial flow-responsive transcription factor Klf2 has been described as the main mechanosensitive transcription factor required for cardiac valve formation (*9*, *10*, *12*, *16*, *18*, *28*–*31*). However, zebrafish *klf2a/b* double mutants only display a partially penetrant valveless phenotype (*12*, *29*, *32*), indicating that other transcription factors play critical roles in transducing the mechanical signals that promote valvulogenesis.

Here, we show that the transcription factor Early growth response 3 (Egr3) is a critical transducer of mechanical signaling during cardiac valve morphogenesis in zebrafish. We first show that loss of *egr3* causes a complete and highly penetrant lack of cardiac valves. Data from loss- and gain-of-function experiments indicate that mechanical forces activate the expression of *egr3* in a subset of EdCs, thereby inducing their migration toward the adjacent ECM. Using transcriptomic analyses followed by functional investigation, we also show that the nuclear receptor Nr4a2b is a target and effector of Egr3 during cardiac valve formation. Furthermore, we show that mechanical forces up-regulate *EGR3* expression in porcine valvular endothelial cells (VECs) and that *EGR3* and its target/effector *NR4A2* may be involved in human cardiac valve remodeling upon ectopic biomechanical overload.

## RESULTS

### Egr3 is a critical regulator of cardiac valve formation

**Fig. 1.**
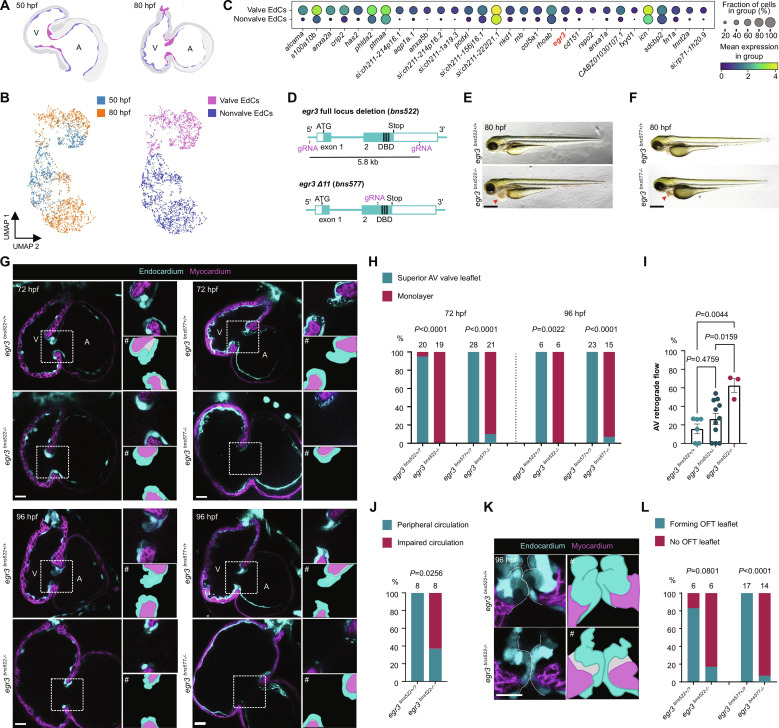
Egr3 is required for cardiac valve formation. (**A**) Schematic of hearts isolated for scRNA-seq; valve EdCs (magenta), nonvalve EdCs (blue). (**B**) scRNA-seq of EdCs from 50 and 80 hpf dissected zebrafish hearts. (**C**) Dot plot of 30 most differentially expressed genes in valve compared with nonvalve EdCs. (**D**) Schematic of generation of *egr3* full locus deletion (*bns522*) and Δ11 (*bns577*) alleles. (**E** and **F**) Brightfield images of *egr3*^+/+^ and *egr3*^−/−^ sibling larvae at 80 hpf; red arrowheads point to pericardial edema. (**G**) Confocal images of representative hearts from 72 and 96 hpf *egr3*^+/+^ and *egr3*^−/−^ sibling larvae. (**H**) Percentage of *egr3*^+/+^ and *egr3*^−/−^ sibling larvae with a superior valve leaflet and without a superior valve leaflet (i.e., endocardial monolayer) at 72 and 96 hpf; seven and four independent experiments, respectively. (**I**) AV retrograde blood flow shown as a fraction of three cardiac cycles; *n* = 6, 11, and 3; one experiment; mean ± SEM, one-way ANOVA followed by Tukey’s post hoc test. (**J**) Peripheral circulation in the caudal vein plexus at 80 hpf, one experiment. (**K**) Confocal images of representative OFT valves from 96 hpf *egr3*^+/+^ and *egr3*^−/−^ sibling larvae. (**L**) Percentage of *egr3*^+/+^ and *egr3*^−/−^ sibling larvae with and without forming OFT leaflets at 96 hpf; four independent experiments. (G and K) EdCs are marked by *Tg(kdrl:*eGFP*)* expression (cyan), and myocardial cells by *Tg(myl7:*BFP-CAAX*)* expression (magenta). # indicates illustrative vectorized cartoons of the valves. (H), (J), and (L) Fisher’s exact test. AV, atrioventricular; V, ventricle; A, atrium; OFT, OFT; DBD, DNA binding domain; EdC, EdC. Scale bars, 400 μm [(E) and (F)] and 20 μm [(G) and (K)].

Seeking to identify additional transcription factors required for cardiac valve formation in zebrafish, we first determined and explored the transcriptional landscape of EdCs at the time when key morphogenetic events underlying valve development take place. Thus, we isolated wild-type zebrafish hearts at 50 hours postfertilization (hpf) when valve identity has just been established, and at 80 hpf when forming valves are first observed (Fig. 1A) (*12*–*14*, *33*). We carried out single-cell RNA sequencing (scRNA-seq) of whole hearts and obtained 1310 and 2194 EdCs at 50 and 80 hpf, respectively (Fig. 1B). The valve population was clearly identified at both time points by the expression of valve endocardium specific genes such as *has2* (*34*) and *alcama* (*33*) (fig. S1A). Differential expression analysis between valve and nonvalve EdCs revealed *early growth response 3* (*egr3*) in the top 30 most enriched genes in the valve cluster and the only one encoding a DNA binding protein with transcription factor activity (Fig. 1C). Notably, *egr3* expression appears more enriched in the valve EdCs when compared with other established valve transcription factor genes (*16*, *18*, *23*, *35*), including *klf2a/b* (*29*) and *nfatc1* (*14*) (fig. S1, B and C). We further analyzed *egr3* expression by in situ hybridization and observed a similarly restricted mRNA localization in the AV canal (fig. S1D). Two other Egr family members have been implicated in cardiac valve development. In mouse, loss of *Egr2* (*Krox20*) results in aortic valve defects and regurgitation (*36*, *37*), while in zebrafish and humans, *egr1*/*EGR1* has been shown to be expressed in cardiac valves (*38*, *39*) and this expression requires mechanical forces (*38*, *40*). We observed *egr1* expression mostly at 80 hpf and in most EdCs (fig. S1, B and C), and neither *egr2a/b* nor *egr4* was detectable in our dataset (fig. S1, B and C). Although the expression pattern and potential role of Egr3 in heart development have not been investigated yet, analyses of human and mouse heart scRNA-seq datasets (*41*, *42*) reveal that *EGR3*/*Egr3* is expressed in cardiac valve endocardium and AV mesenchyme (fig. S2, A and B). Sequence alignment shows that the Egr1 to Egr4 DNA binding domains are highly conserved (fig. S2, C and D), indicating the potential existence of common targets.

Given the strong and early enrichment of *egr3* in the valve endocardium, we investigated its role in valvulogenesis by generating two mutant alleles using the CRISPR-Cas9 system, a 5.8-kb full locus deletion (*bns522*) (i.e., a null allele), and an 11–base pair (bp) deletion (Δ11) (*bns577*), resulting in a premature termination codon that is predicted to disrupt the DNA binding domain (Fig. 1D and fig. S1, E and F)*.* During early development, the valveless embryonic heart achieves unidirectional blood flow in part because of localized contraction waves and a simple vascular network (*43*) whose capacitance provides pressure storage and diminishes cardiac efforts (*44*). By 72 hpf, the AV EdCs have folded into a functional prevalvular structure capable of reducing retrograde blood flow and therefore supporting cardiac output (*10*, *14*, *15*). *egr3* mutants, of both alleles, are indistinguishable from their wild-type siblings until 72 hpf when they start to exhibit noticeable pericardial edema (Fig. 1, E and F). In contrast to their wild-type siblings, *egr3* mutants fail to form AV valve leaflets as their EdCs remain as a monolayer in a fully penetrant fashion in the null allele (19/19) and a nearly fully penetrant fashion in the Δ11 allele (19/21) (Fig. 1, G and H, and fig. S1, G and H). This complete lack of AV valves results in severe retrograde blood flow (Fig. 1I and movies S1 and S2) and leads to heart failure and impaired peripheral circulation (Fig. 1J and movies S3 and S4); *egr3* mutants no longer display cardiac output by 96 hpf (movies S1 to S4). Despite this impaired circulation, cardiac contraction and heart rate are not obviously affected in *egr3* mutants (movies S1 and S2). In 96 hpf wild-type larvae, the AV valves are more mature and elongated and the forming OFT valve leaflets are also present (*14*, *16*, *45*). At this stage, the AV EdCs in *egr3* mutants still remain as a monolayer (Fig. 1, G and H, and fig. S1, G and H) and, similarly, the forming OFT valves also fail to develop (Fig. 1, K and L, and fig. S1I). Together, these data show that Egr3 is essential for cardiac valve formation in zebrafish.

### Endothelial cell–specific deletion of *egr3* results in a lack of AV valves

**Fig. 2.**
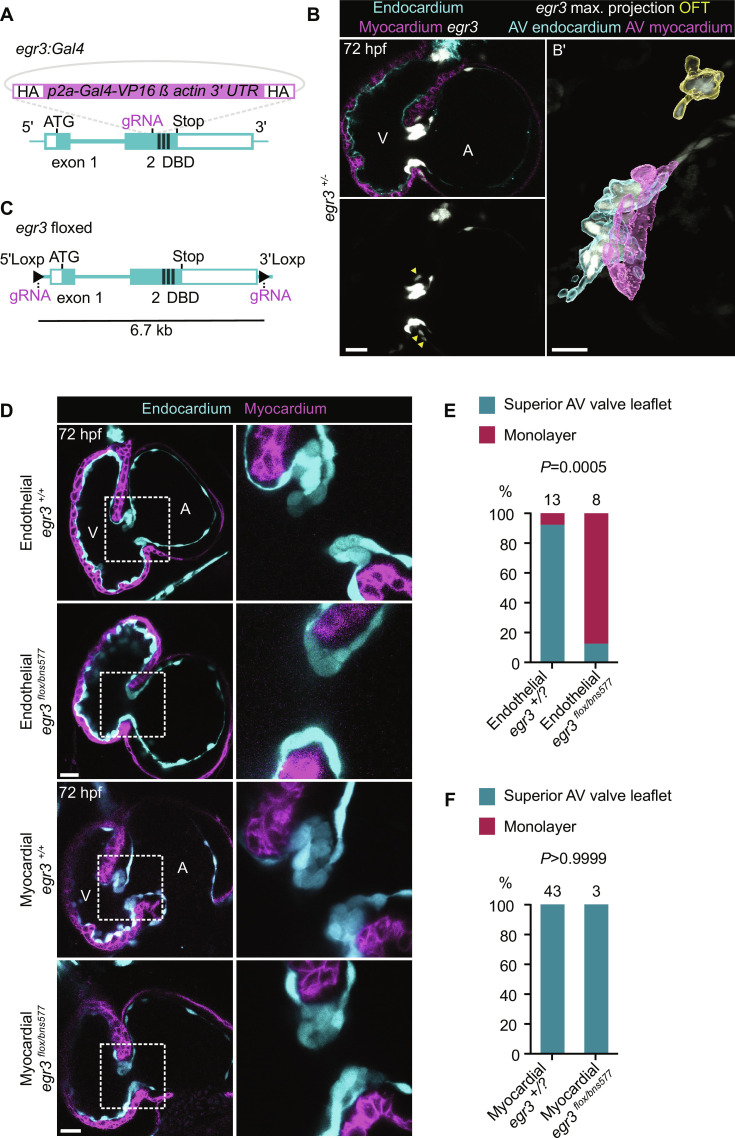
Endothelial-specific deletion of *egr3* recapitulates the global mutant phenotype. (**A**) Schematic of the knock-in reporter [*Pt(egr3:Gal4-VP16*)] generated by inserting *Gal4-VP16* in exon 2 of *egr3*. (**B**) *Pt(egr3:*Gal4-VP16); *Tg(UAS:*eGFP) expression (gray) at 72 hpf; EdCs are marked by *Tg(kdrl:*Hsa.HRAS-mCherry) expression (cyan) and myocardial cells by *Tg(myl7:*BFP-CAAX*)* expression (magenta); yellow arrowheads point to AV canal *egr3* reporter^+^ cardiomyocytes. (**B′**) Maximum projection of *Pt(egr3:*Gal4-VP16); *Tg(UAS:*eGFP) expression with image segmentation of *egr3* reporter*^+^* AV canal EdCs (cyan), *egr3* reporter*^+^* AV canal myocardial cells (magenta), and *egr3* reporter*^+^* OFT cells (yellow). (**C**) Schematic of *egr3* floxed allele *Pt(egr3:loxP-egr3-loxP)*. (**D**) Confocal images of representative hearts from 72 hpf *egr3*^+/+^, endothelial cell–specific *egr3*^−/−^, and myocardial cell–specific *egr3*^−/−^ larvae using the *egr3* floxed allele line. (**E** and **F**) Percentage of 72 hpf *egr3*^+/?^, endothelial cell–specific *egr3*^−/−^ (E), and myocardial cell–specific *egr3*^−/−^ (F) larvae with a superior valve leaflet and without a superior valve leaflet (i.e., endocardial monolayer); one and two independent experiments, respectively; Fisher’s exact test. Scale bars, 20 μm.

To more precisely assess *egr3* expression in the developing heart, we generated a Gal4 knock-in reporter line [*Pt(egr3:Gal4-VP16*)], hereafter referred to as *egr3:Gal4* (*bns576*), by targeting exon 2 using the CRISPR-Cas9 system, thereby disrupting the zinc-finger DNA binding domain (Fig. 2A). Consequently, the *egr3:Gal4* line has endogenous *egr3* reporter activity and is also an *egr3* mutant allele (fig. S3B). *egr3* reporter^+^ embryos display a strong expression in the olfactory bulb as well as in specific structures of the central and peripheral nervous systems (fig. S3A). *egr3* reporter expression is also evident in the presumptive AV canal starting at 34 hpf (fig. S3A), mostly in the AV endocardium, but also in the AV myocardium (Fig. 2B). Moreover, *egr3* reporter expression is observed in the OFT endocardium starting at 72 hpf (Fig. 2B), coinciding with the onset of OFT valve morphogenesis (*16*). Notably, most *egr3* reporter–expressing EdCs were observed in, or near, the AV canal or OFT.

Considering that both endocardial and myocardial cells in the AV canal express *egr3* based on the *egr3:*Gal4 reporter expression, we sought to determine whether *egr3* deletion in one of these cell types was sufficient to recapitulate the *egr3* mutant phenotype. Thus, we used the CRISPR-Cas9 system to generate a sequential *loxP* site knock-in in the *egr3* locus, specifically 440 bp upstream of the transcription start site and 1.9 kb downstream of the stop codon (Fig. 2C). Injection of *Cre* mRNA into embryos from *egr3 ^flox/+^* zebrafish crossed with *egr3^+/−^* zebrafish resulted in 72 hpf larvae displaying pericardial edema and no valve leaflets, correlating with the recombination of the *egr3* floxed allele (fig. S3, D to F). No phenotype was observed in the uninjected larvae, indicating that the *egr3* floxed allele is functional. When crossing *egr3 ^flox/+^* zebrafish with *egr3^+/−^* zebrafish carrying an endothelial Cre driver *(kdrl:Cre)*, we observed that deleting *egr3* in endothelial cells was sufficient to recapitulate the global *egr3* mutant phenotype, thereby resulting in the complete lack of AV valve leaflets (fig. S3G and Fig. 2, D and E). Moreover, when crossing *egr3 ^flox/+^* zebrafish with *egr3^+/−^* zebrafish carrying a myocardial Cre driver (*myl7:Cre*), we observed that deleting *egr3* in myocardial cells led to no noticeable differences with their control siblings including in their valve leaflets (fig. S3H and Fig. 2, D to F). Collectively, these data show that *egr3* functions in the endothelium to drive AV valve formation.

### *egr3* directs AV canal EdC migration through its target Nr4a2b

To gain more insight into the cellular and molecular causes leading to the absence of the AV valve in *egr3* mutants, we analyzed *egr3^+/?^* and *egr3^−/−^* sibling embryos at 48 hpf, a time when they cannot be visually distinguished from each other. At this stage, the AV canal is already morphologically and molecularly distinct from the atrial and ventricular chambers (*33*). Tissue convergence and a decrease in AV canal EdC size are important morphogenetic steps that precede AV valve formation (*46*, *47*). Thus, to evaluate possible morphological differences in the endocardium leading to the *egr3* mutant phenotype, we analyzed EdC volume in each cardiac region (fig. S4, A and B). Consistent with published data (*47*), AV canal EdCs had a smaller volume when compared with ventricular and atrial EdCs (fig. S4C). Notably, there were no differences in EdC cell number or volume in any cardiac region between *egr3* mutants and their *egr3^+/?^* siblings (fig. S4, C and D) such that *egr3* mutant EdCs were observed in expected numbers and underwent the cell shape changes typically observed during early AV valve morphogenesis.

**Fig. 3.**
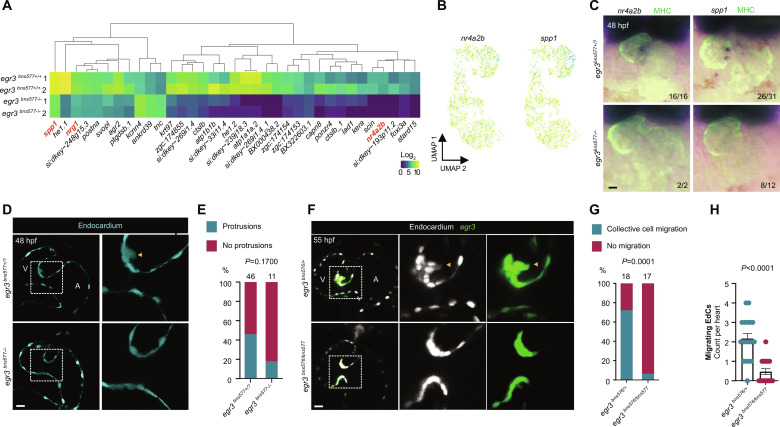
*egr3* is necessary for EdC migration, which is required for cardiac valve formation. (**A**) RNA-seq heatmap analysis of differentially expressed genes; fold change ± 1.5, *P*_adj_ < 0.05, mean > 5. (**B**) scRNA-seq endocardial expression pattern of Egr3 targets *nr4a2b* and *spp1*. (**C**) In situ hybridization for Egr3 targets *nr4a2b* and *spp1* in *egr3*^+/+^ and *egr3*^−/−^ siblings at 48 hpf. (**D**) Confocal images of representative hearts from 48 hpf *egr3*^+/?^ and *egr3*^−/−^ sibling embryos displaying, respectively, AV EdC protrusions toward the ECM and no protrusions; EdCs are marked by *Tg(kdrl:*eGFP*)* expression (cyan). (**E**) Percentage of hearts from 48 hpf *egr3*^+/?^ and *egr3*^−/−^ sibling embryos displaying AV EdC protrusions toward the ECM; three independent experiments. (**F**) Confocal images of representative hearts from 55 hpf *egr3*^+/−^ and *egr3*^−/−^ sibling embryos displaying, respectively, collective AV EdC migration and no migration; EdCs are marked by *Tg(kdrl:*NLS-mCherry*)* expression (gray) and AV EdCs by *Pt(egr3:*Gal4-VP16);*Tg(UAS*:eGFP) expression (green). (**G**) Percentage of hearts from 55 hpf *egr3*^+/−^ and *egr3*^−/−^ sibling embryos displaying AV EdC migration; three independent experiments. (**H**) Number of migrating AV EdCs per heart in 55 hpf *egr3*^+/−^ and *egr3*^−/−^ sibling embryos; *n* = 18 and 17 embryos; three independent experiments. Student’s *t* test, mean ± SEM. (E) and (G) Fisher’s exact test. Scale bars, 20 μm.

To identify the molecular causes of the *egr3* mutant phenotype, we performed bulk RNA-seq analysis of 48 hpf *egr3^+/+^* and *egr3^−/−^* dissected hearts (Fig. 3A). One hundred fifty-four genes were differentially expressed between *egr3* wild-type and mutant sibling embryos. Notably, important valve regulators such as *klf2a/b*, *notch1b*, *dll4*, *nfatc1,* or *wnt9a/b* did not appear to be differentially expressed in *egr3* mutants (fig. S5A). Using in situ hybridization for *egr1*, *has2*, *hey2, klf2a*, *klf4*, *piezo2a*, and immunostaining for Alcama, we observed that the patterning of the AV canal was obviously not affected in *egr3* mutants (fig. S5, B and C), altogether suggesting that lack of *egr3* leads to the absence of the AV valve despite apparently unaffected expression of established cardiac valve markers and regulators. We further screened our dataset for differentially expressed genes with cardiac valve expression and found that *nr4a2b, spp1*, and *nrg1* were significantly down-regulated in *egr3* mutants (Fig. 3A). Nr4a2/Nurr1 is a nuclear receptor with transcription factor activity (*48*), and *nr4a2b* exhibits cardiac valve expression according to our scRNA-seq data (Fig. 3B), while *spp1*/*osteopontin* and *nrg1* are expressed by valve EdCs (Fig. 3, B and C) (*49*, *50*). Our observations that *nr4a2b* and *spp1* are down-regulated in *egr3* mutants agree with published reports in mammals showing that EGR3 is upstream of *Nr4a2* (*51*) and that NR4A2 activates *SPP1* expression by binding to its promoter (*52*). Moreover, EGR3 (*53*, *54*), NR4A2 (*55*), and SPP1 (*56*, *57*) promote endothelial cell migration downstream of vascular endothelial growth factor (VEGF), supporting the model that Egr3 is necessary for EdC migration during valvulogenesis.

To test whether deficient cellular migration could be the cause of the *egr3* mutant phenotype, we evaluated EdC migration in the initial steps of AV valve morphogenesis. From 48 hpf onward, the AV canal EdCs start to extend protrusions into the adjacent ECM and, within a few hours, two to four AV canal EdCs collectively migrate in a ventricle to atrium direction (Fig. 3D) (*13*, *14*). *egr3* mutants exhibit a twofold reduction in the number of AV canal EdC protrusions toward the ECM (Fig. 3E), although this reduction was not significant. Strikingly, the subsequent collective EdC migration is severely affected, as only 6% (*n* = 1/17) of the mutant embryos engaged in EdC migration compared with 72% (*n* = 13/18) of their heterozygous siblings at 55 hpf (Fig. 3, F and G). Between two to four migrating EdCs were observed in these heterozygous hearts, while zero to two migrating EdC was generally observed in these *egr3* mutant hearts (Fig. 3H).

**Fig. 4.**
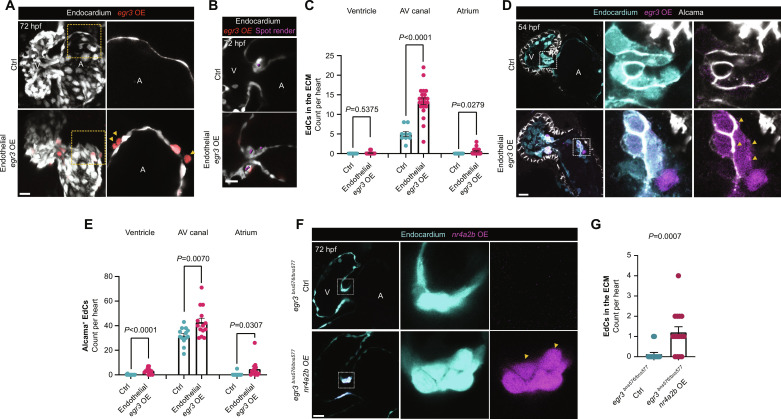
*egr3* triggers EdC migration and Alcama expression. (**A**) 3D reconstruction of representative hearts from control [*Tg(fli1a:Gal4);Tg(UAS:Kaede)*] and endothelial-specific *egr3*-overexpressing [*Tg(fli1a:Gal4);Tg(UAS:Kaede);Tg(UAS:egr3-p2a-dTomato)*] 72 hpf larvae; magnified regions show single *z* planes of the atrium from control and endothelial-specific *egr3*-overexpressing 72 hpf larvae; yellow arrowheads point to *Tg(UAS:*egr3-p2a-dTomato*)*^+^ atrial EdCs in the adjacent ECM. (**B**) Confocal images of representative AV VIC quantification through the spot render function in Imaris; VICs (magenta spots). (**C**) Quantification of EdCs present in the ECM per cardiac region in 72 hpf endothelial-specific *egr3*-overexpressing larvae; *n* = 9 control and 22 *egr3 OE* larvae, *n* = 0 and 0.14 EdCs (ventricle), *n* = 5 and 13.36 EdCs (AV canal), *n* = 0 and 0.59 EdCs (atrium). (**D**) Confocal images of representative hearts from control [*Tg(fli1a:Gal4);Tg(UAS:Kaede)*] and endothelial-specific *egr3*-overexpressing [*Tg(fli1a:Gal4);Tg(UAS:Kaede);Tg(UAS:egr3-p2a-dTomato)*] 54 hpf embryos immunostained for Alcama; magnified regions show Alcama^+^ cells in the AV canal of the control heart and in the atrium of the *Tg(UAS:egr3-p2a-dTomato)* heart. (**E**) Quantification of Alcama^+^ EdCs per cardiac region of control and endothelial-specific *egr3*-overexpressing embryos at 54 hpf; *n* = 13 control and 14 *egr3 OE* embryos, *n* = 0.07 and 3.28 EdCs (ventricle), *n* = 31.77 and 42.86 EdCs (AV canal), *n* = 0.38 and 4.71 EdCs (Atrium); three independent experiments. (**F**) Confocal images of representative hearts from control *Pt(egr3:Gal4-VP16)* and *egr3*-driven *nr4a2b*-overexpressing [*Pt(egr3:Gal4-VP16); Tg(UAS:nr4a2b-p2a-dTomato)*] *egr3*^−/−^ larvae at 72 hpf. (**G**) Quantification of EdCs present in the AV canal ECM of control and *nr4a2b*-overexpressing *egr3*^−/−^ larvae at 72 hpf; *n* = 16 and 15 larvae; two independent experiments. (C), (E), and (G) Student’s *t* test, mean ± SEM. Scale bars, 20 μm.

To investigate whether Egr3 is sufficient to induce EdC migration into the ECM, we generated a stable *egr3* overexpression line, *Tg(5XUAS:egr3-p2a-dTomato)*, hereafter referred to as “*egr3* OE.” To induce ectopic *egr3* overexpression in the endothelium, we crossed *egr3* OE zebrafish with the endothelial *fli1a:Gal4* driver, *Tg(fli1a:Gal4FF). egr3*-overexpressing cells were clearly distinguished by dTomato fluorescence (Fig. 4A). In control hearts, VICs were present in the ECM of the AV canal, but not in the ECM of the ventricle or atrium (Fig. 4, B and C). However, in *egr3* OE hearts, not only was there more than a 2.5-fold increase in VIC number in the ECM of the AV canal (Fig. 4, B and C), but dTomato^+^ EdCs were also present in the ventricular and atrial ECM, adjacent to the endocardial monolayer (Fig. 4, A to C). To better characterize the increased ECM invasion by EdCs in *egr3* OE hearts, we assessed the expression of the endocardial valve marker Alcama (*33*). Notably, *egr3* overexpression in the endothelium was sufficient to induce ectopic Alcama expression in ventricular and atrial EdCs (Fig. 4, D and E), as well as a significant increase of Alcama-expressing EdCs in the AV canal (Fig. 4E). Next, we tested whether overexpressing the Egr3 target, Nr4a2b, would be sufficient to rescue AV canal EdC migration in *egr3* mutants. Thus, we generated a stable UAS-driven *nr4a2b* overexpression line, *Tg(5XUAS:nr4a2b-p2a-dTomato*), hereafter referred to as “*nr4a2b* OE,” and used it in the background of the *egr3 bns577* mutant allele and *egr3:Gal4^+/−^*. The resulting transheterozygous progeny fail to produce a functional Egr3 protein and overexpress *nr4a2b* in *egr3:*Gal4^+^ cells. Transheterozygous larvae displayed the expected *egr3* mutant phenotype at 72 hpf (Fig. 4F and fig. S3B). *egr3* transheterozygous larvae overexpressing *nr4a2b* in their AV canal endocardium displayed a significant increase in the number of VICs in the AV canal ECM, indicating a partial rescue of EdC migration (Fig. 4, F and G). Not all VICs in *nr4a2b* OE hearts were dTomato*^+^*, indicating that Nr4a2b may also act in a cell nonautonomous fashion in this process. As previously reported for *klf2a* overexpression in the AV canal, using an *nfatc:Gal4* line (*15*), we found that *nr4a2b* has an inhibitory effect on valve formation in *egr3*^+/−^ larvae (fig. S5, D and E), suggesting that *nr4a2b* expression needs to be tightly regulated during valvulogenesis. Collectively, these data indicate that the absence of cardiac valves in *egr3* mutants is a result of failed EdC migration into the adjacent ECM and that Egr3 is sufficient to induce endocardial Alcama expression and migration; they also suggest that Egr3 promotes AV canal EdC migration through its target Nr4a2b.

### *egr3* is a mechanosensitive transcription factor gene in cardiac valve development and disease

**Fig. 5.**
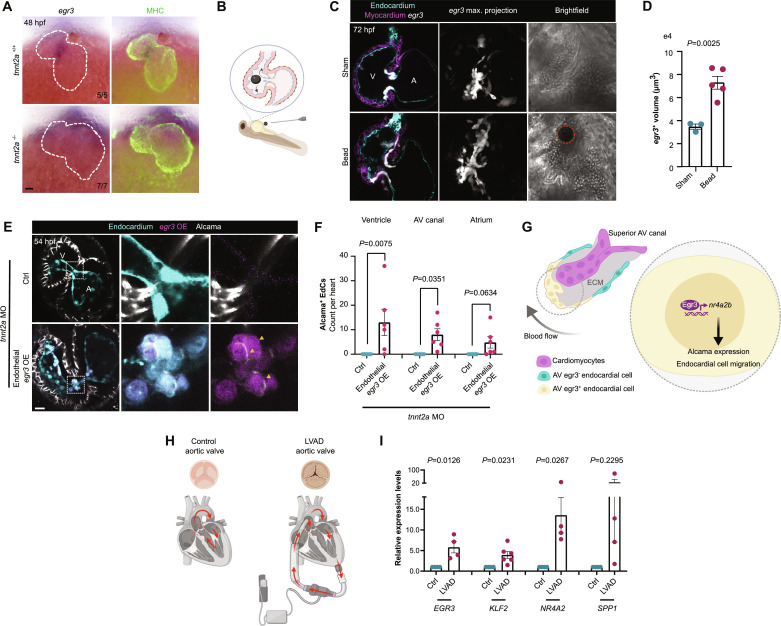
*egr3* expression in the heart is regulated by biomechanical forces. (**A**) In situ hybridization for *egr3* expression in 48 hpf *tnnt2a*^+/+^ and *tnnt2a*^−/−^ sibling embryos. (**B**) Illustration of bead insertion into a 48 hpf heart. (**C**) *Pt(egr3:*Gal4-VP16); *Tg*(*UAS:*eGFP) expression (gray) at 72 hpf in bead-inserted larva versus sham; myocardial cells are marked by *Tg(myl7:*BFP-CAAX*)* expression (magenta) and EdCs by *Tg(kdrl:*Hsa.HRAS-mCherry) expression (cyan). Red dotted circle highlights inserted bead; yellow arrowhead points to *egr3^+^* cells near the bead. (**D**) Quantification of total volume of *egr3* reporter^+^ cells in bead-inserted larvae and sham; *n* = 3 sham and 5 experimental larvae; one experiment. (**E**) Confocal images of representative hearts from control [*Tg(fli1a:Gal4);Tg(UAS:Kaede)*] and endothelial-specific *egr3*-overexpressing [*Tg(fli1a:Gal4);Tg(UAS:Kaede);Tg(UAS:egr3-p2a-dTomato)*] *tnnt2a* MO–injected embryos immunostained for Alcama at 54 hpf; magnified regions show the AV canal of *tnnt2a* MO–injected embryos with rescued Alcama expression in the *Tg(UAS:egr3-p2a-dTomato)* embryo. (**F**) Quantification of Alcama^+^ EdCs per cardiac region of *tnnt2a* MO–injected embryos at 54 hpf; *n* = 6 control and 6 *egr3 OE* embryos, *n* = 0.00 and 8.00 EdCs (ventricle), *n* = 0.00 and 13.00 EdCs (AV canal), *n* = 0.00 and 4.83 EdCs (Atrium); two independent experiments. (**G**) Oscillatory shear stress induces the expression of the mechanosensitive transcription factor gene *egr3* in valve EdCs, where it orchestrates valvulogenesis by promoting Alcama expression and their migration via Nr4a2b. (**H**) Schematic of aortic valve collection from LVAD patients and control donors. (**I**) Relative mRNA levels of *EGR3*, *KLF2*, *NR4A2*, and *SPP1* from aortic valves of LVAD patients and control donors; *n* = 4 and 4 to 6; one experiment. Ct values can be found in data S1. (D), (F), and (I) Student’s *t* test, mean ± SEM. (B) and (H) Illustrations were generated with Biorender.com. Scale bars, 20 μm.

Considering that EGR3 is involved in mediating VEGF-induced endothelial migration (*53*) and that it is a VEGF target via extracellular signal–regulated kinase (ERK) activation (*58*), we decided to test whether *egr3* AV canal expression was dependent on VEGF signaling. We found that *egr3* AV canal expression appeared to be unaltered after treatment with VEGF receptor inhibitors or a mitogen-activated protein kinase kinase (MEK) inhibitor (fig. S6A), suggesting that it is independent of VEGF/ERK signaling. Given the fundamental role of mechanical signaling in cardiac valve formation (*10*, *23*, *26*), we then decided to test whether *egr3* expression was downstream of mechanical forces. We simulated a no-flow/no-contraction condition in vivo by taking advantage of the zebrafish embryo’s ability to withstand severe cardiac dysfunction (*59*). We used *silent heart* zebrafish mutants, which lack the sarcomeric protein Tnnt2a and, therefore, do not display cardiac contraction (*59*). Notably, *egr3* AV canal expression was completely abrogated in *tnnt2a* mutant hearts (Fig. 5A), and we also observed the loss of *egr3* expression when treating zebrafish embryos with the myosin inhibitor BDM (fig. S6A). To test whether *egr3* expression responds to ectopic mechanical stimulation, we performed microsurgical insertion of a bead inside the embryonic heart through the inflow tract (*22*, *60*) at 48 hpf (Fig. 5B). The bead remained inside the heart for 24 hours and moved rhythmically across the ventricular chamber following the heartbeats and without stopping the blood flow. Upon this ectopic mechanical stimulation, *egr3* expression was expanded in the hearts of the bead-inserted larvae when compared with sham controls, as shown by the expression of the *egr3:Gal4* reporter (Fig. 5, C and D). We did not find ectopic EdCs in the ECM or an increased number of VICs in the bead inserted hearts when compared with sham controls (fig. S6B). As mechanical forces are required for valve formation, we next tested whether overexpressing *egr3* in endothelial cells in no-flow conditions could rescue some of the early steps of valve development. We injected *tnnt2a* morpholinos (MOs) into one-cell stage embryos of *egr3* OE zebrafish crossed with the endothelial *fli1a:Gal4* driver (Fig. 5E). Valvular cell identity is absent in no-flow condition hearts as the endocardium remains as a monolayer and the expression of valve markers is absent (Fig. 5E) (*26*, *61*). Notably, some *egr3*-overexpressing endothelial cells in no-flow condition hearts expressed Alcama, displayed a partly cuboidal valvular cell shape, and were often found in the ECM (Fig. 5E). These Alcama^+^ *egr3*-overexpressing cells were present in the endocardium of both the ventricle and atrium as well as in the AV canal of no-flow condition hearts (Fig. 5F and fig. S6C), suggesting a lack of spatial specification of valve identity in no-flow hearts and/or the ability of Egr3 to drive this specification on its own.

To investigate whether *EGR3* is activated in mammalian VECs in response to shear stress, we used a unique fluid activation device specifically designed to expose cells to physiologically relevant pulsatile wall shear stress (WSS) conditions (*62*). We observed an up-regulation of *EGR3* expression in porcine VECs exposed to pulsatile WSS compared with static conditions (fig. S6D), indicating that *EGR3* responds to pulsatile shear stress in VECs. In addition to playing an important role in valvulogenesis, mechanical forces are also pivotal during pathological valve remodeling. For instance, the use of a left ventricular assist device (LVAD) has become an established treatment option as well as a bridge for end-stage heart failure patients awaiting a heart transplant (*63*). Despite improving survival and heart function, LVAD use is correlated with aortic insufficiency, and 15 to 52% of patients may present worsening of previous aortic valve pathology or develop de novo aortic valve insufficiency (*64*). The LVAD increases cardiac output by diverting the blood from the left ventricle straight into the aorta, thereby decreasing the strain in the mitral valve and causing biomechanical overload in the aortic root and valves (*65*). This increased biomechanical overload in the aortic valves induces valve remodeling (i.e., aortic valve fusion) (*66*, *67*). However, how the aortic valves transduce the biomechanical overload into a molecular response remains elusive. As zebrafish *egr3* expression responds to lack of, as well as increase in, mechanical forces during valve development (Fig. 5, A to D) and *EGR3* responds to pulsatile WSS in VECs (fig. S6D), we sought to investigate whether *EGR3* expression was altered during human aortic valve remodeling following LVAD placement. We collected samples of aortic valves from control donors and LVAD patients and performed quantitative reverse transcription polymerase chain reaction (RT-qPCR) (Fig. 5H). *EGR3* expression was increased by almost sixfold in LVAD patient valves compared with donor control valves (Fig. 5I). Expression of the classical mechanosensitive transcription factor gene *KLF2* was increased by nearly fourfold (Fig. 5I). We also tested EGR3 targets and observed that in LVAD patient valves, *NR4A2* expression was increased by more than 13-fold and *SPP1* expression by more than 20-fold, although the latter was not significant (Fig. 5I).

## DISCUSSION

Here, we identify the transcription factor Egr3 as a master transducer of the mechanical forces that guide valvulogenesis in zebrafish, and show that Egr3 is required for EdC migration into the adjacent ECM by activating the expression of the migration regulator *nr4a2b* (*55*). *Klf2* has long been considered to be the main mechanosensitive transcription factor gene in cardiac valve formation. However, the broad endocardial expression of *klf2a/b* compared with the restricted expression of *egr3* in valve EdCs suggests that Egr3 has a more specific role in transducing mechanical forces during valvulogenesis. *klf2* mutations in zebrafish result in a 47 to 56% penetrance of the cardiac valveless phenotype observed in no-flow conditions (*29*, *32*). Moreover, *klf2* mutants also display cardiomyocyte extrusion (*32*) and it is not clear how this phenotype affects cardiac valve formation. In contrast, the *egr3* mutant phenotype is highly penetrant and it is specific to the valves, with no obvious defects in other cardiac regions.

*EGR3* is expressed in AV canal EdCs and fibroblasts in mammalian hearts (*41*, *68*), and it responds to pulsatile WSS in VECs, consistent with a conserved function in valvulogenesis. *Egr3* mutant mice display an increased frequency of perinatal mortality (*69*), and it would be worth investigating them for any underlying cardiac defects. In humans, *EGR3* single-nucleotide variants resulting in loss-of-function alleles are highly underrepresented with a loss-of-function observed/expected upper bound fraction of 0.396 (gnomAD v4.0.0), possibly suggesting that *EGR3* is haploinsufficient. Haploinsufficiency is also typically observed in genes associated with bicuspic aortic valve disease, such as *GATA6* (*70*) and *NOTCH* (*71*, *72*). Of note, case reports have shown that chromosomal duplications of the region where *EGR3* is located (8p21.3) are associated with congenital heart disease (i.e., valve and septal defects) (*73*, *74*), while a case-control study has reported an association between an *EGR3* locus polymorphism and coronary artery disease (CAD) (*75*), though with no mention of possible valve defects.

In parallel to *egr3* up-regulation following mechanical stimulation in the developing zebrafish heart, *EGR3* is up-regulated in porcine VECs and in LVAD aortic valves upon biomechanical overload. In addition, *EGR3* expression does not seem to respond to fluid shear stress in human umbilical vein endothelial cells (HUVECs) (*76*), suggesting a cell- and/or flow-specific response. In addition, EGR3 is necessary and sufficient to partially mediate the transforming growth factor–β (TGF-β) fibrotic response in fibroblasts (*77*). Thus, we speculate that the biomechanical stimuli that promote *EGR3* expression during cardiac development also promote it in LVAD aortic valves, thereby contributing to pathological aortic valve remodeling and insufficiency (*65**–**67*). It will be important to investigate the role of *EGR3*, *KLF2,* and other mechanosensitive transcription factor genes in LVAD aortic valve pathogenesis.

Transduction of mechanical forces, which is mediated in part by VEGF signaling (*78*), also plays a role in lymphatic endothelial cell (LEC) development and physiology. VEGF is a known regulator of *EGR3* and *NR4A2* expression in endothelial cells (*53*, *58*, *79*). Human LECs up-regulate *EGR3* and *NR4A2* expression following VEGF stimulation (*80*, *81*), and a recent report on the transcriptional coactivator Zmiz1 suggests that *Egr3* is also involved in lymphatic valve formation (*82*).

In summary, our study reveals a previously unknown signaling axis in which EGR3 is necessary to transduce the mechanical signals required for cardiac valve formation and potentially also for LVAD valve remodeling. We anticipate that perturbations in the function or expression of *EGR3*, or its targets, also lead to cardiac valve defects in humans.

## MATERIALS AND METHODS

### Zebrafish handling and lines

All zebrafish husbandry was performed under standard conditions in accordance with institutional (Max Planck Gesellschaft) and national (German) ethical and animal welfare regulations. All procedures performed on animals conform to the guidelines from Directive 2010/63/EU of the European Parliament on the protection of animals used for scientific purposes and were approved by the Animal Protection Committee (Tierschutzkommission) of the Regierungspräsidium Darmstadt (reference: B2/1218). The following mutant, knock-in reporter, and transgenic lines were generated for this study: *egr3^bns522^*, *egr3^bns577^*, *Pt(egr3:Gal4-VP16)^bns576^* abbreviated as *egr3*:*Gal4*, *Pt(egr3:loxP-egr3-loxP)^bns661^*, *Tg(5xUAS:egr3*-*p2a-dTomato)^bns607^* abbreviated as *egr3*:*OE*, and *Tg(5xUAS:nr4a2b*-*p2a-dTomato)^bns719^* abbreviated as *nr4a2b*:*OE.* The following transgenic and mutant lines were used in this study: *tnnt2a^mn0031Gt^ or Gt(GBT-R14)* (*83*)*, Tg(kdrl:EGFP)^s843^* (*33*)*, Tg(kdrl:Hsa.HRAS-mCherry)^s896^* (*84*)*, Tg(kdrl:NLS-mCherry)^is4^* (*85*)*, Tg(fli1a:Gal4FF)^ubs4^* (*86*)*, Tg(myl7:BFP-CAAX)^bns193^* (*87*)*, Tg(myl7:mCherry-CAAX)^bns7^* (*88*)*, Tg(myl7:EGFP)^twu26^* (*89*), *Tg(5xUAS:EGFP)^nkuasgfp1a^* (*90*)*, Tg(UAS:Kaede)^rk8^* (*91*)*, Tg(myl7:Cre)^sd55^* (*92*)*, Tg(kdrl:Cre)^s898^* (*93*), and *Tg(Mmu.Hhex-E1B:GFP)^bns321^* (*14*)*.*

### Generation of zebrafish lines

For CRISPR-Cas9 mutagenesis, in vitro synthesis of Cas9 mRNA and design of guide RNAs (gRNAs) were performed as previously described (*94*, *95*). gRNA efficiency was evaluated in single AB wild-type injected embryos by high-resolution melting analysis (HRMA) or T7 endonuclease assay. Generation of *egr3* full locus deletion (*bns522*) was achieved by using one gRNA in the immediate 5′ intergenic region and another in the 3′ UTR, and F1 adults were screened by PCR followed by sequencing of PCR products. The *egr3* Δ11 allele (*bns577*) was generated with commercially available *egr3* gRNA [Integrated DNA Technologies (IDT)], and F1 adults were screened by HRMA followed by sequencing of PCR products. The *egr3:Gal4* reporter line (*bns576*) was generated according to the GeneWeld method (*96*) with 48-bp homology arms flanking the *p2a-Gal4-VP16-beta-actin* sequence, and using the *egr3* gRNA (IDT) as for the *egr3* Δ11 allele (*bns577*). We used the previously published sequential *loxP* knock-in method (*97*) to generate the *egr3* floxed allele (*bns661*) with single-stranded donor oligonucleotides containing *loxp* sites flanked by asymmetric 21- and 49-bp homology arms. For the 5′ *loxP* knock-in, we used the same 5′ gRNA as for the full locus deletion allele (*bns522*). Adult F1 zebrafish were screened for the 5′ *loxP* integration using a *loxP*-specific PCR primer and sequenced after performing a *loxP*-flanking PCR. The 5′ *loxP* founder zebrafish was then crossed with AB wild type, and the resulting one-cell stage embryos were injected with a 3′ gRNA designed to cut the neighboring intergenic region. Adult F1 *egr3* floxed allele zebrafish were screened by *loxP*-specific and flanking PCRs followed by sequencing of PCR products. Despite observing a complete and adequate integration of the 5′ *loxP* site, we have not been able to sequence the full extent of the 3′ *loxP* site. Genetic polymorphisms and/or integration of multiple copies after mutagenesis of the 3′ region in the zebrafish used for 3′ *loxP* knock-in might explain why we could not sequence the *loxP* integration. However, the floxed *egr3* allele was confirmed by the *loxP*-specific and flanking PCRs, as well as by functional analysis after *Cre* mRNA injection, in which a recombined band of 400 bp was identified by PCR and the *egr3* phenotype was observed at the expected Mendelian ratios. Conditional knockouts were obtained by crossing *egr3^flox/+^* zebrafish with *egr3^bns577/+^* zebrafish carrying *Tg(kdrl:Cre)* or *Tg(myl7:Cre)*, for endothelial or myocardial cell–specific knockout, respectively. The low number of myocardial cell–specific knockout larvae (3 of 46) (Fig. 2, D and F) is under the expected ratio of 1:8 for a cross of *egr3^bns577/+^*, *egr3^flox/+^*, and *myl7:Cre^+/−^* zebrafish and without selecting the progeny for a phenotype. We used *tol2* transgenesis to generate the *egr3* (*bns607*) and *nr4a2b* (*bns719*) overexpression lines, respectively *egr3:OE* and *nr4a2b:OE*. Adult *egr3:OE and nr4a2b:OE* F1 zebrafish were screened by crossing with *Tg(fli1a:Gal4FF)* zebrafish and using the dTomato^+^ expression in the progeny as a proxy. dTomato^+^ embryos were used to confirm transgenesis by PCR amplification and sequencing of the PCR products. Of note, the data presented in this work were generated using progeny from F1 to F3 animals (obtained from sequential outcrosses to different transgenic lines or nontransgenic AB zebrafish); while doing the revisions, we observed a reduction in the penetrance of the cardiac valve phenotype in the progeny of F4 animals. Genotyping PCRs were performed with KAPA2G Fast Ready Mix (Sigma-Aldrich 2GFRMKB) and HRMAs with Maxima SYBR Green/Fluorescein qPCR Master Mix (2X) (Thermo Fisher Scientific K0241). The sequences of all genotyping primers, CRISPR sites, and donor oligonucleotides are available in data S3.

### Plasmid construction

The open reading frames (ORFs) for *egr3*, *nr4a2b*, *spp1*, *nrg1*, *hey2,* and *has2* were amplified from cDNA generated from a pool of 78 hpf larvae, and cloned into a pCS2+ vector. To generate the *egr3:Gal4* line, the *pGTag-Gal4-VP16-beta-actin* plasmid was a gift from J. Essner (Addgene # 117817) and homology arms were added by vector ligation (*98*). To generate the *egr3:OE* line (*bns607*) and *nr4a2b:OE* line *(bns719)*, *egr3* and *nr4a2b* were amplified from pCS2+ and cloned into a *5XUAS:p2a-dTomato* vector. Vector ligation was performed by PCR amplification followed by in vivo cloning (*99*). All primers are listed in data S3.

### Microscopy

Live imaging of stopped zebrafish hearts was performed by mounting N-Phenylthiourea (PTU)-treated embryos and larvae in 1% agarose containing 0.2% tricaine. We used the following confocal microscopes: LSM700 Axio Imager 2 and LSM880 Axio Examiner with a W Plan-Apochromat 40×/1.0 dipping lens for image acquisition. We used Fiji (ImageJ 2.1.0/1.53o) to adjust the brightness and contrast of representative images. For three-dimensional (3D) rendering and analysis of confocal images, we used Imaris x64 (Bitplane, 10.0.1). We used the Imaris cell identification function to automatically segment EdCs based on their nucleus, marked by *Tg(kdrl:*NLS-mCherry*)* expression, and their cytoplasm, marked by *Tg(kdrl:*EGFP*)* expression. The EdCs were further segregated into ventricular, AV, and atrial with the help of the *x*/*y* position object filtering followed by manual curation. Illustrative cartoons were created using Inkscape vector graphics editor unless otherwise indicated in the figure legends.

### In situ hybridization and immunostaining

Whole-mount RNA in situ hybridization was performed as previously described (*100*). In situ probes for *egr3*, *spp1*, *nrg1*, *hey2*, and *has2* were synthesized from previously cloned ORFs in pCS2+ vectors using PCR products with an incorporated T7 promoter. An in situ probe for *egr1* was provided by J. Vermot in a pBSK vector. In situ probes for *klf2a* (*15*), *klf4, nr4a2b*, and *piezo2a* (*15*) were synthesized directly from a PCR product of 78 hpf wild-type AB cDNA, using primers with an incorporated T7 promoter. To visualize the cardiac outline, we used the primary mouse anti-MHC (myosin heavy chain) antibody [1:500, MF-20, Developmental Studies Hybridoma Bank (DSHB) AB_2147781], followed by the secondary goat anti-mouse immunoglobulin G (IgG) Alexa Fluor 488 antibody (1:500, Invitrogen A11029). To assess AV valve identity, we performed immunostaining for Alcama using mouse anti-Alcama antibody (1:50, ZN-8, DSHB AB_531904) followed by goat anti-mouse Alexa Fluor 647 antibody (1:500, Invitrogen A21236). In situ hybridizations were imaged using an SMZ25 stereomicroscope (Nikon) with a 2×/0.3 objective. The sequence of primers used to synthesize the in situ probes can be found in data S3.

### AV blood flow fraction

Functional analysis of blood regurgitation was performed in 72 hpf zebrafish larvae mounted in 1% agarose without tricaine. The zebrafish hearts were imaged live using an inverted Cell Observer Spinning Disk microscope with a 25×/0.8 water-immersion objective at 240 frames per second (fps). Quantification of AV blood flow fraction was performed using a custom-made ImageJ script as previously described (*15*). For each imaged larva, AV retrograde blood flow is shown as a fraction of three averaged cardiac cycles.

### Microsurgical insertion of a bead

To assess the influence of ectopic mechanical stimulation on *egr3* expression in vivo, a bead was kept inside of the beating zebrafish heart for 24 hours (*22*, *60*). For that manipulation, 48 hpf zebrafish embryos were mounted ventrally in 1% agarose and a microsurgical incision was performed in the yolk sac using thin forceps. A bead (Cube Biotech 32201) was inserted with the forceps into the yolk cavity and gently pushed into the inflow tract, eventually reaching the heart through suction. Embryos were released from the agarose and kept in egg water at 28.5°C for 24 hours, when *egr3:*Gal4; *UAS:*eGFP expression was assessed by confocal microscopy. Only larvae that had the bead inside of the ventricle and still displayed blood flow were considered for the analysis. Sham embryos underwent the same procedures of mounting and yolk piercing, but without bead insertion.

### Chemical treatments

To evaluate the influence of cardiac contraction on *egr3* expression, we treated wild-type embryos with 15 mM of the myosin inhibitor BDM (Sigma-Aldrich B0753). Possible regulation of AV *egr3* expression by VEGF signaling was tested by treating embryos with 1 and 2.5 μM SKLB1002 (Selleck Chemicals S7258) (*101*) and 0.5 μM SU5416 (Sigma-Aldrich S8442). Possible regulation of AV *egr3* expression by ERK signaling was tested by treating embryos with 1 μM of the MEK inhibitor PD0325901 (Sigma-Aldrich PZ0162) (*2*). Concentrations were adjusted to diminish side effects. Dimethyl sulfoxide (DMSO; 0.1%) was administered as vehicle, and all treatments were performed from 36 to 48 hpf, when embryos were collected and fixed for in situ hybridization.

### Porcine aortic valve endothelial cell isolation and culture

We used a fluid activation device that applies physiologically relevant pulsatile WSS on the surface of porcine aortic VECs as previously described (*62*). VECs were isolated from porcine aortic valve leaflets. Briefly, aortic valve leaflets were isolated and incubated in collagenase type II/Dulbecco’s modified Eagle medium (DMEM) (Life Technologies) for 3 × 7 min. After incubation, cells were gently scraped to isolate VECs from the two sides of the leaflets. After isolation, VECs were seeded and cultured on flask coated with rat collagen type I (50 μl/ml) (10 mg/ml concentration, BD Biosciences). VECs were cultured at 37°C and 5% CO_2_ in Endothelial Cell Growth Medium (Promocell) supplemented with manufacturer’s adjuvants and 5% fetal bovine serum (FBS). After 1 hour of culture, VECs were first removed from the collagen gels and lysed in TRIzol (Thermo Fisher Scientific 15596026). RNAs were extracted using RNeasy Mini Kit (Qiagen 74104).

### Research ethics for donated aortic valve samples

The study population consisted of LVAD (*n* = 6) patients referred for heart transplantation at La Timone Hospital (*102*). Patients were not included if they had connective tissue disease and previous aortic valve dysfunction. The patients provided their written informed consent to participate in this study (approved by the Marseille ethics committee n°13.061). Aortic regurgitation (AR) was graded through an integrative approach (*103*, *104*). Mechanism of AR was separated into prolapse of the fused leaflet, cusp restriction, or both. Aortic valves were recognized by their anatomical landmarks under the microscope, and leaflets were isolated with minimal aortic wall contamination. Immediately following surgical removal, a portion of the aortic valve leaflet was placed in RNAlater solution (Sigma-Aldrich) and stored at −80°C until processing. Control valve samples were obtained at autopsy of individuals without cardiac problems who suffered a traumatic death (*n* = 8). The control group was composed of individuals with an average age of 59 ± 4 years, and a male to female ratio (M/F) of 85%. The LVAD group was composed of individuals with an average age of 54 ± 12 years, and M/F ratio of 71%. This study was performed according to the principles of the Declaration of Helsinki and in accordance with institutional guidelines.

### Quantitative RT-qPCR

Total RNA from human aortic valve samples was purified using TRIzol (Life Technologies) and RNeasy Mini Kit. RNA was reverse-transcribed using the AffinityScript Multiple Temperature cDNA synthesis kit (Agilent). Quantitative PCR was performed using SYBR Green (Roche)–based qPCR on a LightCycler 480 (Roche). Graphs show the mean ± SD for six to eight biological replicates. Samples were normalized to *TBP* or *GAPDH* as endogenous housekeeping genes. Relative expression levels were calculated by the comparative cycle threshold (ΔΔCt) method. All Ct and ΔCt values are listed in data S1. The sequence of primers used for RT-qPCR can be found in data S3.

### scRNA-seq sample preparation and data analysis

To obtain a longitudinal transcriptional signature of single EdCs, we isolated hearts from *Tg(myl7:EGFP)* wild-type zebrafish at 50 and 80 hpf. Up to 150 hearts were isolated per condition as previously described (*105*). Briefly, zebrafish embryos and larvae were fragmented using a needle and syringe in an Eppendorf tube with 1 ml of DMEM (Thermo Fisher Scientific 88281) with 10% FBS (Sigma-Aldrich F2442). The homogenate was filtered through a 100-μm mesh filter. An additional wash with DMEM with 10% FBS was performed to remove the remaining hearts in the Eppendorf tube, followed by filtering. The isolated hearts were manually collected from the flow-through under a fluorescence microscope. The hearts were further dissociated using the Pierce Primary Cardiomyocyte Isolation Kit (Thermo Fisher Scientific 88281) and incubated for 25 min at 30°C (shaker 300 rpm) with pipetting every 5 to 10 min. Dead cells were removed from the final cell isolate by fluorescence-activated cell sorting in phosphate-buffered saline (PBS) without calcium or magnesium (Lonza, 17-516F) and with 0.04% bovine serum albumin (BSA). The cell suspensions were counted with a Moxi cell counter and diluted according to manufacturer’s protocol to obtain 10,000 single-cell data points per sample. Each sample was run separately on a lane in a Chromium controller with Chromium Next GEM Single Cell 3′ Reagent Kits v3.1 (10x Genomics). scRNA-seq library preparation was done using a standard protocol, and sequencing was done on Nextseq2000. scRNA-seq analysis was performed as previously described (*106*), and final data visualization was done using a CellxGene package (doi:10.5281/zenodo.3235020). EdCs were identified by the expression of endothelial markers (e.g., *cdh5*, *fli1a*, *kdrl*, and *tie1*) and represented 3504 from the 6476 sequenced heart cells. Valve EdCs were identified by the expression of valve markers (e.g., *alcama* and *has2*) and represented 1343 cells. Data analysis was performed using only the EdCs, and the other cell types are not displayed for visualization purposes. The classification for transcription factor used was based on the Gene Ontology (GO) terms containing direct DNA binding domain, such as “DNA-binding transcription factor activity, RNA polymerase II-specific” and “DNA binding.” Thus, *crip2* did not appear in our gene enrichment analysis as the encoded protein seems to lack a direct DNA binding domain (*107*).

### RNA-seq sample preparation and data analysis

To further investigate the *egr3* mutant phenotype, we conducted a bulk RNA-seq analysis of dissected zebrafish hearts at 48 hpf. As *egr3* mutants cannot be distinguished from their siblings at this stage, embryos were live genotyped at 36 hpf as previously described (*108*). We dissected 20 hearts for each biological duplicate of *egr3^bns577−/−^* and *egr3^bns577+/+^* sibling samples in cold DMEM with 10% FBS. Total RNA was isolated using the miRNeasy Micro Kit (Qiagen 217084), followed by on-column deoxyribonuclease (DNase) digestion (DNase-Free DNase Set, Qiagen 79254). Final elution was performed in 12 μl of ribonuclease (RNase)–free water, and subsequent RNA quality control, cDNA preparation, sequencing, and analyses were performed as previously described (*101*). A total of 154 genes were significantly dysregulated based on *P*_adj_ < 0.05. Heatmaps were obtained using the Webbased Interactive Omics visualizatioN—Applications (WIlsON) (*109*).

### Statistical analysis

GraphPad Prism (v.9) was used to perform all statistical analyses. We used two-tailed Student’s *t* test for comparing two samples (Figs. 3H, 4, C, E, and G, and 5, D, F, and I, and figs. S4D and S6D), and one-way analysis of variance (ANOVA) followed by Tukey’s post hoc test for multiple comparisons (Fig. 1I and fig. S4C); data are presented as mean ± SEM, and actual *P* values are shown. Two-sided Fisher’s exact test was used to compare categorical data between two groups (Figs. 1, H, J, and L, 2, E and F, and 3, E and G, and figs. S3C and S5E), and data were represented as percentages after statistical analysis to improve visualization. To allow for statistical analysis, the classifications “Superior valve leaflet” and “Superior s-shape leaflet” were collapsed into “Superior valve leaflet,” and the “Monolayer / one VIC” and “Monolayer” were collapsed into “Monolayer” (Fig. 1H); all classifications and proportions are in figs. S1 (G to I) and S3I. All genetic experiments with zebrafish were performed independently at least twice, using different batches of embryos and on different days unless otherwise indicated. Exact sample sizes and *P* values are described in the figures and figure legends. *P* values <0.05 were considered significant.
