# Supplementary material for: egr3 is a mechanosensitive transcription factor gene required for cardiac valve morphogenesis
Source: Sci Adv. 2024 May 15;10(20):eadl0633. doi: 10.1126/sciadv.adl0633 (PMC11095463; doi:10.1126/sciadv.adl0633)
Supplement: Supplementary file 1 — Figs. S1 to S6 Legends for movies S1 to S4 Legends for data S1 to S3 [file sciadv.adl0633_sm.pdf]

Science Advances
AAAS

Supplementary Materials for

egr3 is a mechanosensitive transcription factor gene required for cardiac valve morphogenesis

Agatha Ribeiro da Silva *et al.*

Corresponding author: Thomas Juan, thomas.juan@mpi-bn.mpg.de; Didier Y. R. Stainier, didier.stainier@mpi-bn.mpg.de

Sci. Adv. **10**, eadl0633 (2024)
DOI: 10.1126/sciadv.adl0633

The PDF file includes:

Figs. S1 to S6
Legends for movies S1 to S4
Legends for data S1 to S3

Other Supplementary Material for this manuscript includes the following:

Movies S1 to S4
Data S1 to S3

Fig. S1

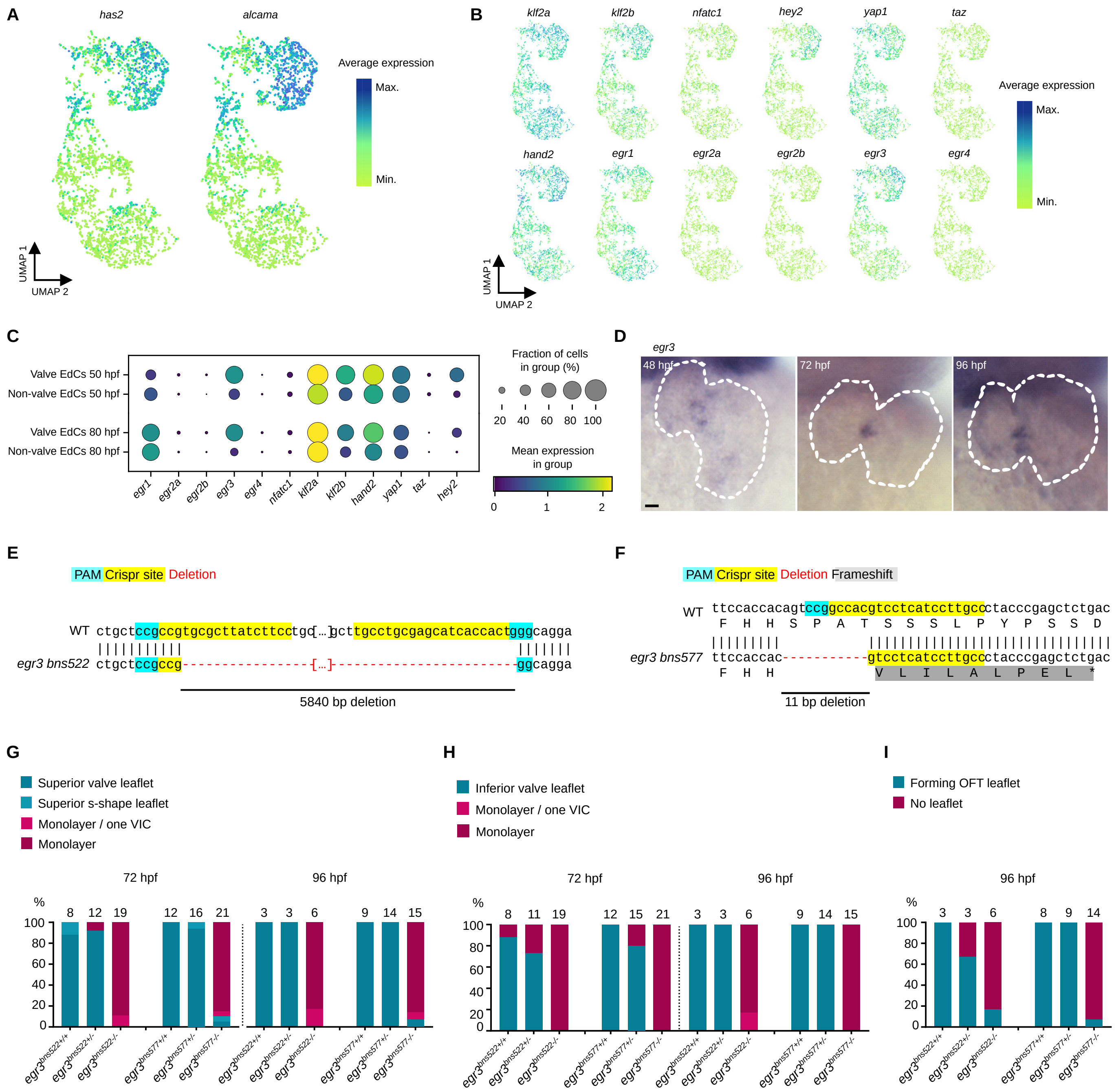
A
has2
alcama
Average expression
Max.
Min.
UMAP 1
UMAP 2
B
klf2a
klf2b
nfatc1
hey2
yap1
taz
hand2
egr1
egr2a
egr2b
egr3
egr4
Average expression
Max.
Min.
C
Valve EdCs 50 hpf
Non-valve EdCs 50 hpf
Valve EdCs 80 hpf
Non-valve EdCs 80 hpf
egr1 egr2a egr2b egr3 egr4 nfatc1 klf2a klf2b hand2 yap1 taz hey2
Fraction of cells in group (%)
20 40 60 80 100
Mean expression in group
0 1 2
D
egr3
48 hpf
72 hpf
96 hpf
E
PAM Crispr site Deletion
WT ctgctccgccgtgcgcttatcttcctgc[...]gcttgcctgcgagcatcaccactgggcagga
egr3 bns522 ctgctccgccg[...]ggcagga
5840 bp deletion
F
PAM Crispr site Deletion Frameshift
WT ttccaccacagtccggccacgtcctcatccttgccctacccgagctctgac
F H H S P A T S S S L P Y P S S D
egr3 bns577 ttccaccacgtcctcatccttgccctacccgagctctgac
F H H V L I L A L P E L *
11 bp deletion
G
Superior valve leaflet
Superior s-shape leaflet
Monolayer / one VIC
Monolayer
72 hpf
96 hpf
H
Inferior valve leaflet
Monolayer / one VIC
Monolayer
72 hpf
96 hpf
I
Forming OFT leaflet
No leaflet
96 hpf

Fig. S1. ***egr3*** **expression is enriched in valve endocardial cells.** (**A**), scRNA-seq endocardial average expression pattern of valve markers *has2* and *alcama*. (**B**), Endocardial expression pattern of key transcription factor genes; *egr3* expression is enriched in the valve endocardium. (**C**), Dot plot of key transcription factor genes in valve and non-valve endocardial cells at 50 and 80 hpf. (**D**), *in situ* hybridization for *egr3* expression at 48, 72 and 96 hpf. (**E-F**), Schematic of *egr3* full locus deletion (*bns522*) and Δ11 (*bns577*) mutant alleles. (**G-I**), Percentage of larvae without or with a superior (**G**), inferior (**H**), and OFT (**I**), valve leaflet phenotype at 72 and 96 hpf; 7 and 4 independent experiments, respectively, (related to Fig. 1H, L). Scale bar = 20 μm.

Fig. S2

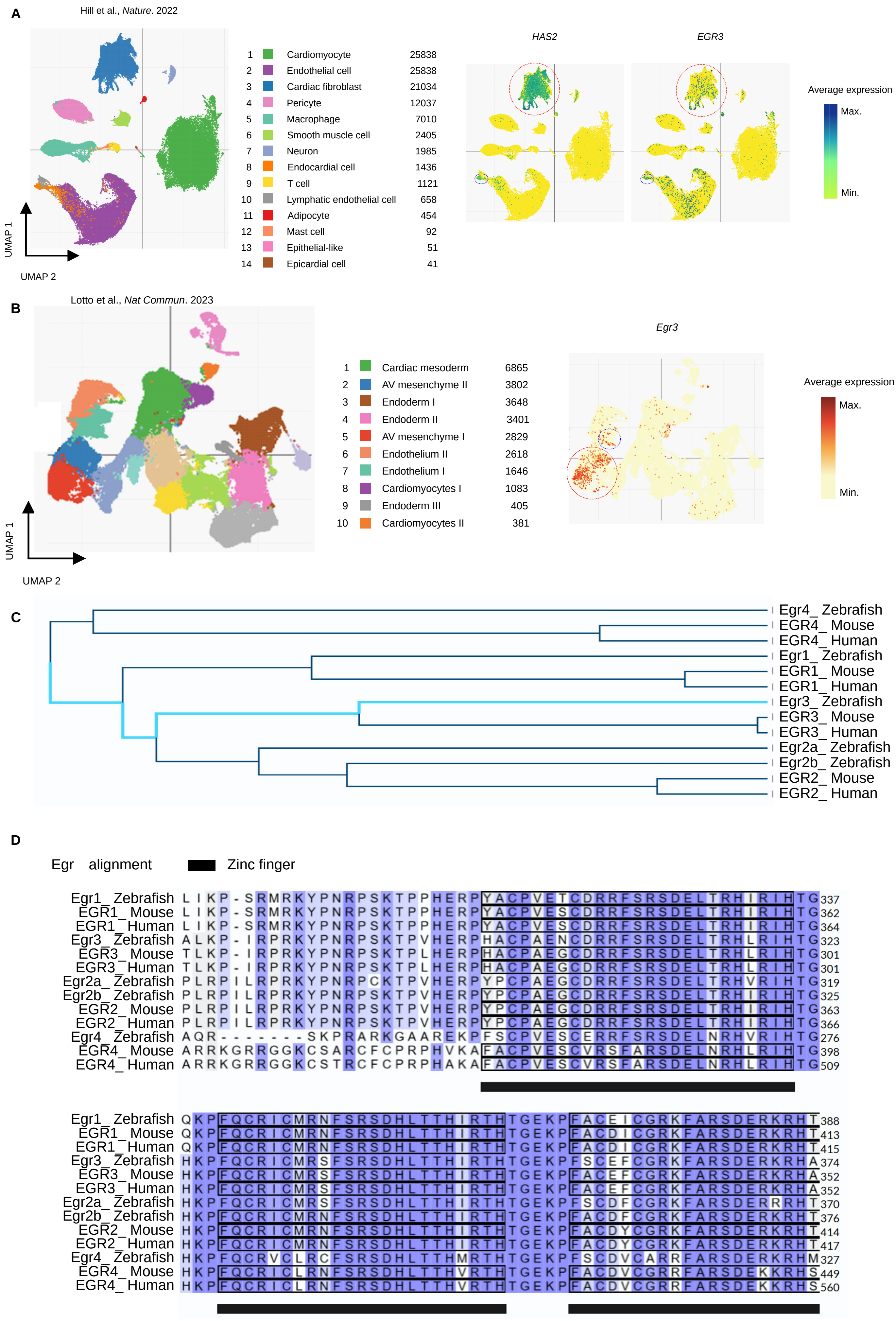

A
Hill et al., Nature. 2022
1 Cardiomyocyte 25838
2 Endothelial cell 25838
3 Cardiac fibroblast 21034
4 Pericyte 12037
5 Macrophage 7010
6 Smooth muscle cell 2405
7 Neuron 1985
8 Endocardial cell 1436
9 T cell 1121
10 Lymphatic endothelial cell 658
11 Adipocyte 454
12 Mast cell 92
13 Epithelial-like 51
14 Epicardial cell 41
UMAP 1
UMAP 2
HAS2
EGR3
Average expression
Max.
Min.
B
Lotto et al., Nat Commun. 2023
1 Cardiac mesoderm 6865
2 AV mesenchyme II 3802
3 Endoderm I 3648
4 Endoderm II 3401
5 AV mesenchyme I 2829
6 Endothelium II 2618
7 Endothelium I 1646
8 Cardiomyocytes I 1083
9 Endoderm III 405
10 Cardiomyocytes II 381
UMAP 1
UMAP 2
Egr3
Average expression
Max.
Min.
C
Egr4_ Zebrafish
EGR4_ Mouse
EGR4_ Human
Egr1_ Zebrafish
EGR1_ Mouse
EGR1_ Human
Egr3_ Zebrafish
EGR3_ Mouse
EGR3_ Human
Egr2a_ Zebrafish
Egr2b_ Zebrafish
EGR2_ Mouse
EGR2_ Human
D
Egr alignment
Zinc finger
Egr1_ Zebrafish 337
EGR1_ Mouse 362
EGR1_ Human 364
Egr3_ Zebrafish 323
EGR3_ Mouse 301
EGR3_ Human 301
Egr2a_ Zebrafish 319
Egr2b_ Zebrafish 325
EGR2_ Mouse 363
EGR2_ Human 366
Egr4_ Zebrafish 276
EGR4_ Mouse 398
EGR4_ Human 509
Egr1_ Zebrafish 388
EGR1_ Mouse 413
EGR1_ Human 415
Egr3_ Zebrafish 374
EGR3_ Mouse 352
EGR3_ Human 352
Egr2a_ Zebrafish 370
Egr2b_ Zebrafish 376
EGR2_ Mouse 414
EGR2_ Human 417
Egr4_ Zebrafish 327
EGR4_ Mouse 449
EGR4_ Human 560

Fig. S2. *EGR3* cardiac valve expression is conserved across species. (**A**), scRNA-seq of adult human heart (41). *EGR3* is expressed in cardiac valve populations of endocardial cells (blue circle) and cardiac fibroblasts (red circle). (**B**), scRNA-seq of mouse heart cells at E7.75, 8.25, 9.25, 10.5, and 12.5 (42). *Egr3* is expressed in the AV mesenchyme (red circle) and endocardial (blue circle) populations. (**C**), Phylogenetic tree of human, mouse, and zebrafish Egr family members. (**D**), Protein alignment of zinc finger DNA binding domain of human, mouse, and zebrafish Egr family members. AV = atrioventricular.

Fig. S3

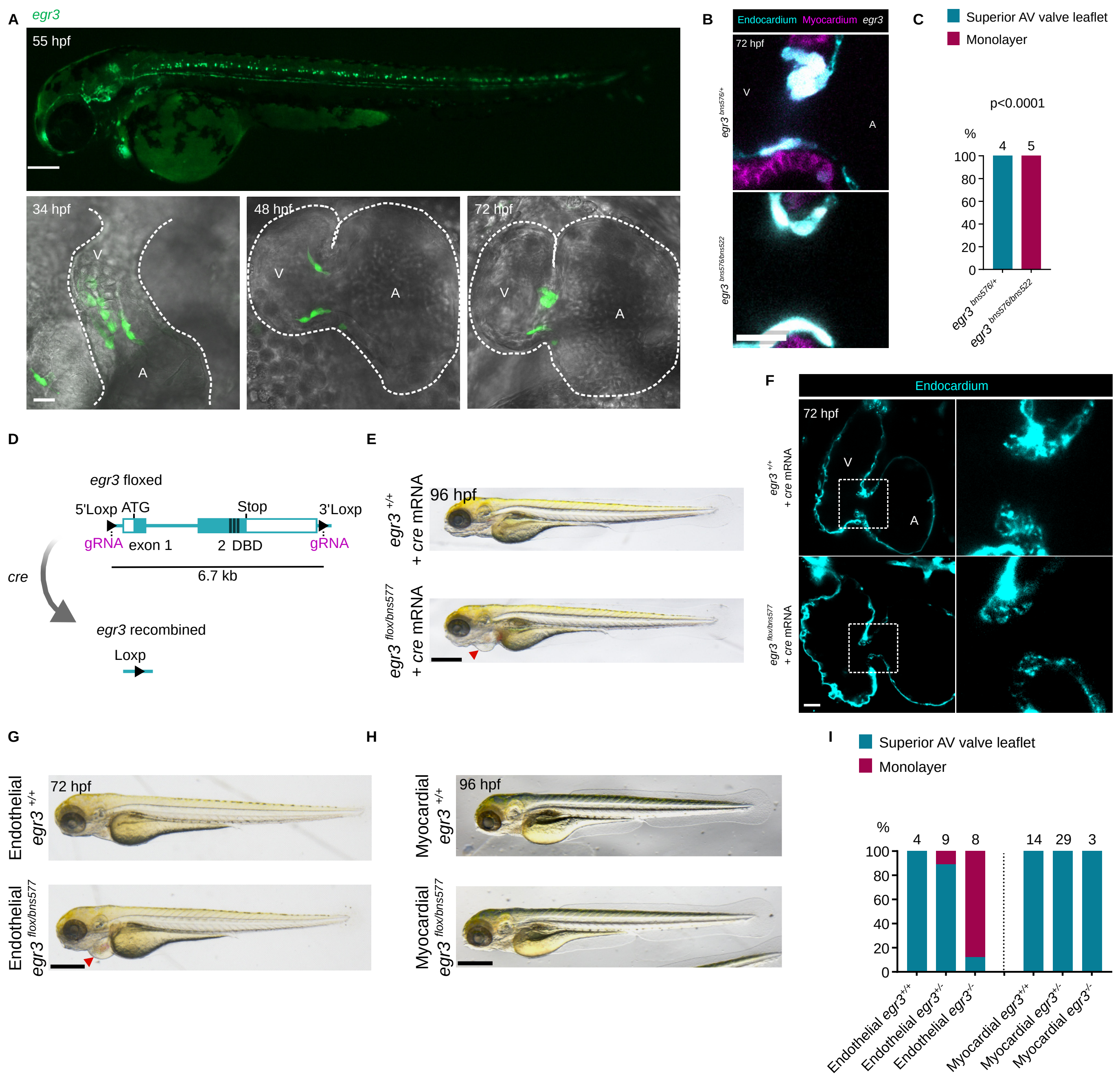
A
egr3
55 hpf
34 hpf
48 hpf
72 hpf
V
A
B
Endocardium Myocardium egr3
72 hpf
egr3 bns576/+
egr3 bns576/bns522
C
Superior AV valve leaflet
Monolayer
p<0.0001
%
4
5
100
80
60
40
20
0
D
egr3 floxed
5'Loxp ATG
Stop
3'Loxp
gRNA
exon 1
2 DBD
6.7 kb
cre
egr3 recombined
Loxp
E
96 hpf
egr3 +/+ + cre mRNA
egr3 flox/bns577 + cre mRNA
F
Endocardium
72 hpf
G
Endothelial egr3 +/+
Endothelial egr3 flox/bns577
H
Myocardial egr3 +/+
Myocardial egr3 flox/bns577
I
Superior AV valve leaflet
Monolayer
%
4
9
8
14
29
3
Endothelial egr3+/+
Endothelial egr3+/-
Endothelial egr3-/-
Myocardial egr3+/+
Myocardial egr3+/-
Myocardial egr3-/-

Fig. S3. Cell-specific role of Egr3. (**A**), *Pt(egr3:*Gal4-VP16*); Tg(UAS:*eGFP*)* expression in the whole embryo at 55 hpf, and in the heart at 34, 48 and 72 hpf. (**B**), Confocal images of representative hearts from 72 hpf $egr3^{bns576/+}$ and $egr3^{bns576/bns522}$ sibling larvae. (**C**), Percentage of $egr3^{+/-}$ and $egr3^{-/-}$ sibling larvae with a superior valve leaflet and without a superior valve leaflet (i.e., endocardial monolayer) at 72 hpf; 1 experiment. Fisher's exact test. (**D**), Schematic of *egr3* floxed allele recombination. (**E**), Brightfield images of *cre* mRNA injected 72 hpf $egr3^{+/+}$ and $egr3^{flox/bns577}$ sibling larvae. (**F**), Confocal images of representative *cre* mRNA injected 72 hpf $egr3^{+/+}$ and $egr3^{flox/bns577}$ sibling larvae; endocardial cells are marked by *Tg(kdrl:*Hsa.HRAS-mCherry) expression (cyan). (**G-H**), Brightfield images of $egr3^{+/+}$, endothelial-specific $egr3^{-/-}$ (**G**), and myocardial-specific $egr3^{-/-}$ (**H**), sibling larvae at 72 and 96 hpf, respectively. **(I)** Percentage of larvae with or without a superior AV valve leaflet (i.e., endocardial monolayer) at 72 hpf; 1 and 2 independent experiments (related to Fig. 2E, F). AV = atrioventricular, V = ventricle, A = atrium. Scale bars (**A** (whole embryo), **E**, **G**, **H**) = 400 μm, (**A** (hearts), **B**, **F**) = 20 μm.

Fig. S4

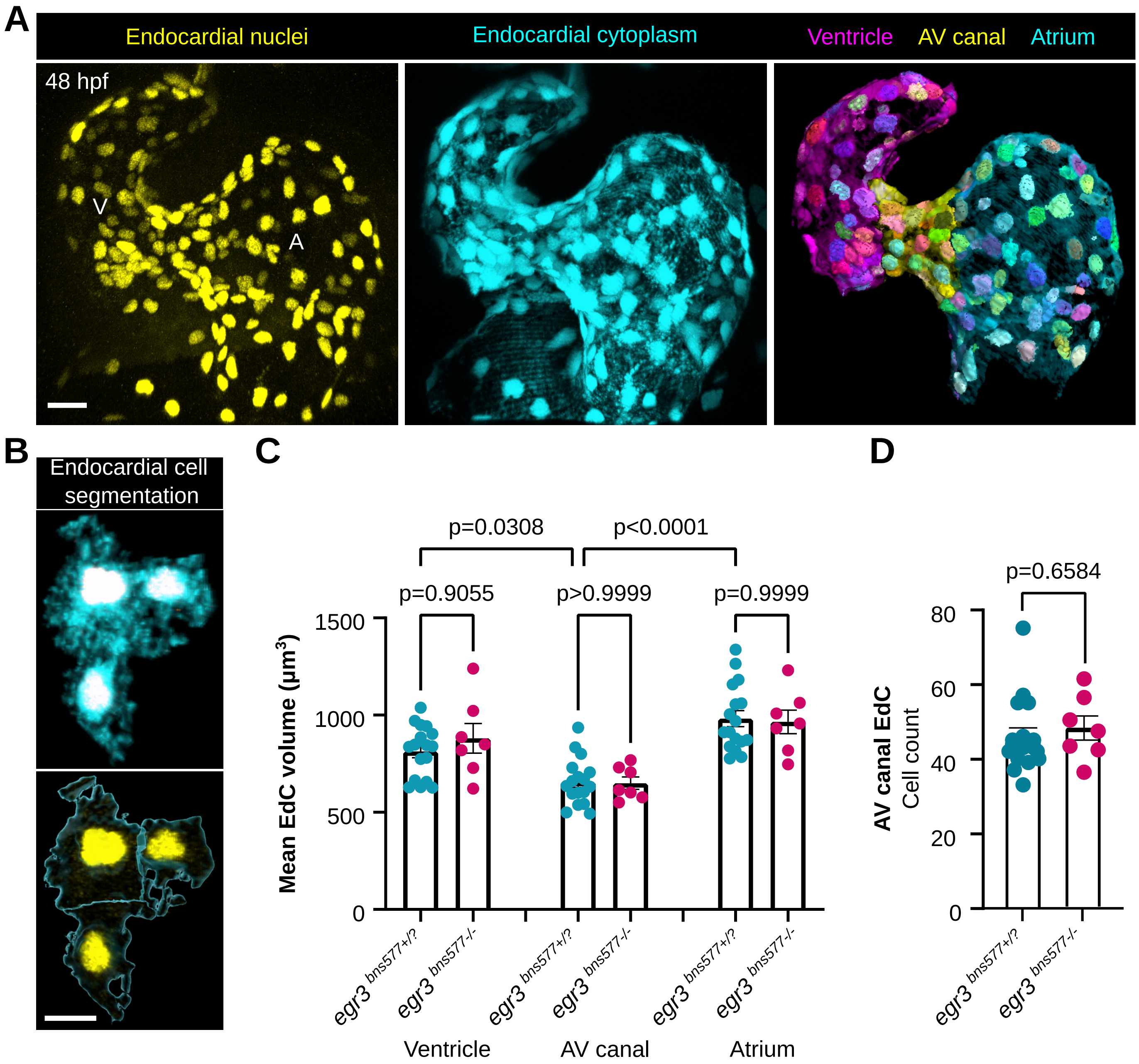
A
Endocardial nuclei
Endocardial cytoplasm
Ventricle
AV canal
Atrium
48 hpf
V
A
B
Endocardial cell segmentation
C
p=0.0308
p<0.0001
p=0.9055
p>0.9999
p=0.9999
Mean EdC volume (µm³)
1500
1000
500
0
egr3 bns577+/?
egr3 bns577-/-
Ventricle
AV canal
Atrium
D
p=0.6584
AV canal EdC
Cell count
80
60
40
20
0

Fig. S4. Endocardial cell morphology is not altered in *egr3* mutants at the onset of the atrioventricular valve phenotype. (**A**), 3D reconstruction of a zebrafish heart at 48 hpf. Endocardial cells are marked by *Tg(kdrl:*NLS-mCherry*)* and *Tg(kdrl:*eGFP*)* expression, respectively showing their nuclei (yellow) and cytoplasm (cyan); individual cells were segmented using Imaris and assigned to the corresponding cardiac regions. (**B**), Individual segmented endocardial cells. (**C**), Average endocardial cell volume per heart in each defined region; mean ± SEM, one-way ANOVA followed by Tukey's post hoc test. (**D**), Number of AV endocardial cells in 48 hpf *egr3*$^{+/+}$ and *egr3*$^{-/-}$ embryos; Student's t test, mean ± SEM. (**C, D**), n = 5, 12, and 7 embryos; 1 experiment. AV = atrioventricular, V = ventricle, A = atrium. Scale bars (**A**) = 20 µm, (**B**) = 10 µm.

Fig. S5

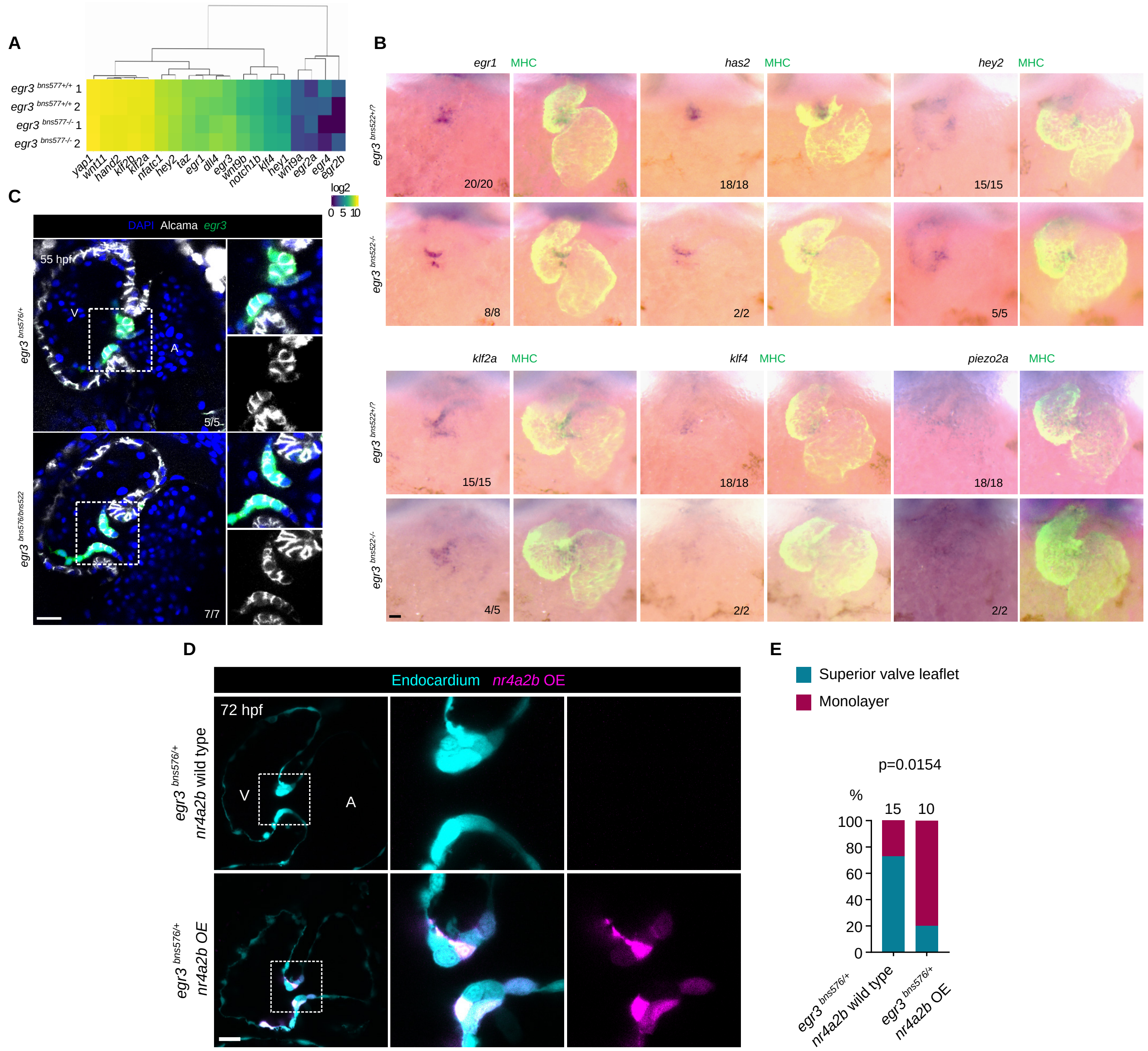

A
B
C
D
E
log2
0 5 10
DAPI Alcama egr3
55 hpf
V
A
5/5
7/7
egr1 MHC
has2 MHC
hey2 MHC
klf2a MHC
klf4 MHC
piezo2a MHC
20/20
18/18
15/15
8/8
2/2
5/5
15/15
18/18
18/18
4/5
2/2
2/2
Endocardium nr4a2b OE
72 hpf
Superior valve leaflet
Monolayer
p=0.0154
%
15
10

Fig. S5. ***egr3*** **mutants display wild-type like patterning of the atrioventricular canal.** (**A**), RNA-seq heatmap analysis of key valve regulator genes. (**B**), *in situ* hybridization for different AV valve markers in 48 hpf *egr3*$^{+/+}$ and *egr3*$^{-/-}$ embryos. (**C**), Confocal images of representative Alcama immunostaining of hearts from 55 hpf *egr3*$^{bns576/+}$ and *egr3*$^{bns576/bns522}$ sibling embryos. **(D)**, Confocal images of representative hearts from control *Pt(egr3:Gal4-VP16)* and *egr3*-driven *nr4a2b* overexpressing (*Pt(egr3:Gal4-VP16); Tg(UAS:nr4a2b-p2a-dTomato)*) *egr3*$^{bns576/+}$ larvae at 72 hpf. **(E)**, Percentage of control and *egr3*-driven *nr4a2b* overexpressing *egr3*$^{bns576/+}$ larvae with and without a superior AV valve leaflet (i.e., endocardial monolayer) at 72 hpf; n = 15 and 10; 2 independent experiments. Fisher's exact test. V = ventricle, A = atrium. Scale bars = 20 µm.

Fig. S6

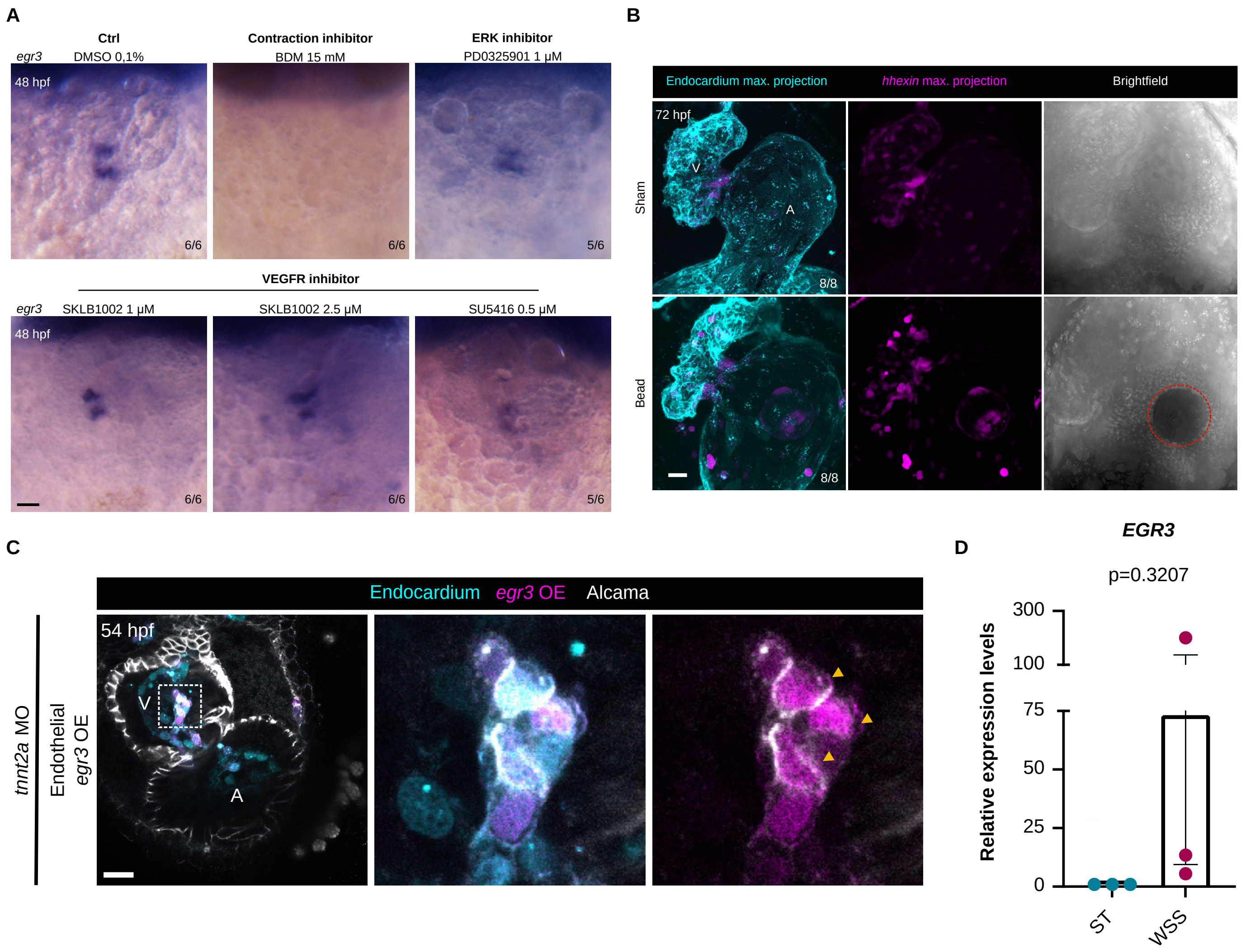
A
Ctrl
Contraction inhibitor
ERK inhibitor
egr3
DMSO 0,1%
BDM 15 mM
PD0325901 1 µM
48 hpf
6/6
6/6
5/6
VEGFR inhibitor
egr3
SKLB1002 1 µM
SKLB1002 2.5 µM
SU5416 0.5 µM
48 hpf
6/6
6/6
5/6
B
Endocardium max. projection
hhexin max. projection
Brightfield
72 hpf
Sham
Bead
V
A
8/8
8/8
C
Endocardium egr3 OE Alcama
54 hpf
tnnt2a MO
Endothelial egr3 OE
V
A
D
EGR3
p=0.3207
Relative expression levels
300
100
75
50
25
0
ST
WSS

Fig. S6. Regulation of *egr3* cardiac valve expression. (**A**), *in situ* hybridization for *egr3* expression following different chemical treatments. (**B**), Confocal images of representative hearts from sham control and bead-inserted larvae at 72 hpf; valve interstitial cells are marked by *Tg(Mmu.Hhex*-E1B:GFP*)* expression (magenta) and endocardial cells by *Tg(kdrl:*Hsa.HRAS-mCherry) expression (cyan). Red dotted circle highlights inserted bead. (**C**), Confocal images of representative hearts from an endothelial-specific *egr3* overexpressing (*Tg(fli1a:Gal4);Tg(UAS:Kaede);Tg(UAS:egr3-p2a-dTomato)) tnnt2a* MO injected embryo immunostained for Alcama at 54 hpf; magnified region shows Alcama e in the ventricle of a *tnnt2a* MO injected *Tg(UAS:egr3-p2a-dTomato)* embryo. (**D**), Relative *EGR3* mRNA levels in porcine aortic valve endothelial cells (PAVECs) subjected to static (ST) or pulsatile wall shear stress (WSS) conditions; n = 3; 1 experiment. Student's t test, mean ± SEM. Ct values can be found in Data S1. **(A, B and C)** Scale bars = 20 µm.

Movie S1. Lack of *egr3* results in severe retrograde blood flow. (**A**), Brightfield live imaging (4.13 msec/frame) of the heart from a representative 80 hpf *egr3*$^{+/+}$ larva. The movie depicts the AV flow profile (anterograde, no-flow, and retrograde) and was saved at 76.4 frames per second (fps) to enhance visualization. Scale bars = 20 µm.

Movie S2. Lack of *egr3* results in severe retrograde blood flow. (**B**), Brightfield live imaging (4.13 msec/frame) of the heart from a representative 80 hpf *egr3*$^{-/-}$ larva. The movie depicts the AV flow profile (anterograde, no-flow, and retrograde) and was saved at 76.4 frames per second (fps) to enhance visualization. Scale bars = 20 µm.

Movie S3. *egr3* mutants display impaired peripheral circulation. (**A**), Brightfield live imaging of the caudal vein plexus from a representative 80 hpf *egr3*$^{+/+}$ larva; 100 frames per second (fps).

Movie S4. *egr3* mutants display impaired peripheral circulation. (**B**), Brightfield live imaging of the caudal vein plexus from a representative 80 hpf *egr3*$^{-/-}$ larva; 100 frames per second (fps).

Data S1. Raw data underlying graphs presented in the figures.

Data S2. Analyzed Bulk RNA-seq of 48 hpf dissected hearts.

Data S3. Lists of oligos and CRISPR sites.
